# Associations of short-term exposure to air pollution with respiratory hospital admissions in Arak, Iran

**DOI:** 10.1186/s40201-017-0277-z

**Published:** 2017-07-17

**Authors:** Mostafa Vahedian, Narges Khanjani, Moghaddameh Mirzaee, Ali Koolivand

**Affiliations:** 1 Neurology Research Center, Kerman University of Medical Sciences, Kerman, Iran; 2 Environmental Health Engineering Research Center, Kerman University of Medical Sciences, Kerman, Iran; 3 Modeling in Health Research Center, Institute for Futures Studies in Health, Kerman University of Medical Sciences, Kerman, Iran; 4 Department of Environmental Health Engineering, Faculty of Health, Arak University of Medical Sciences, Arak, Iran; 5 Department of Epidemiology and Biostatistics, School of Public Health, Kerman University of Medical Sciences, Kerman, Iran

**Keywords:** Air pollution, Respiratory hospital admissions, Time-series regression

## Abstract

**Background:**

Ambient air pollution, is one of the most frequently stated environmental problems. Many epidemiological studies have documented adverse health effects for ambient air pollution. This study aimed to investigate the association between ambient air pollution and respiratory hospital admissions.

**Methods:**

In this ecological time series study data about air pollutant concentrations including CO, NO_2_, O_3_, PM_2.5_, PM_10_ and SO_2_ and, respiratory hospital admissions in the urban population of Arak, from January 1st 2010 to December 31st 2015; were inquired, from the Arak Department of Environment, and two major hospitals, respectively. Meteorological data were inquired for the same period as well. Time-series regression analysis with a distributed lag model, controlled for seasonality long-time trends, weather and day of the week, was used for data analysis.

**Results:**

Every 10 μg/m^3^ increase in NO_2_, and PM_10_ and every 1 mg/m^3^ increase in CO at lag 0 corresponded to a RR = 1.032 (95%CI, 1.003–1.06), RR = 1.01 (95%CI, 1.004–1.017) and RR = 1.09 (95%CI, 1.04–1.14), increase in respiratory disease hospitalizations, respectively. Males and the elderly were found to be more susceptible than females and other age groups to air pollutants in regard to respiratory disease admissions.

**Conclusions:**

The results of this study showed that outdoor air pollutants significantly increase respiratory hospital admissions; especially among the men and elders in Arak.

## Background

Ambient air pollution, which has been exacerbating over the last few decades in the world, is one of the most frequently stated environmental problems, especially in the developing countries [1–4]. This global public health concern was estimated to cause 3.7 million rural and urban premature deaths worldwide in 2012 [5]. Many epidemiological studies have documented increase in outdoor air pollution concentrations associated with adverse health effects, including increased respiratory hospital admission [4, 6–11].

Previous published studies in the world have shown that air pollutants are related to cardiac and respiratory deaths and hospital admissions [12–15]. The adverse effects of increasing air pollution, indicated as an increase in respiratory hospital admissions has been reported from North America and Europe [13–19], and relatively fewer studies of this kind have been conducted in developing countries and the Middle East. For example, a study from Italy found a positive association for PM_10_, SO_2_, NO_2_ and CO with respiratory disease hospital admissions [15]. In another study in two northern New England cities, an interquartile range (IQR) increase in SO_2_ and O_3_ were associated with increase in all respiratory and asthmatic emergency room (ER) visits, in Portland. However, no significant associations between air pollution and respiratory ER visits were found in Manchester, UK [19].

Some studies conducted in Asia have also found a positive relation between air pollutants and respiratory hospital admission [20–24]. For example, one study in Beijing, showed that an increase in NO_2_, PM_10,_ and SO_2_ were associated with an increase in respiratory disease emergency admissions [1]. Another study in Tehran, Iran found that an increase in PM_2.5_, NO_2_, CO, and O_3_, were respectively associated with an increase in respiratory hospital admissions [23].

However, the findings of developed countries might not be generalizable to developing countries because of the different constituents of ambient air pollution or the different demographics in their communities [25]. In developing countries, air pollution is increasingly becoming a major healthcare issue because of increased motor vehicles, traffic, lack of appropriate control on pollutant resources, industrialization [26] and lack of proper legislations. In these countries air pollution has had an ascending trend [26, 27].

Iran, is a developing country, [27], experiencing demographic and epidemiological transition and environmental pollution issues. These changes are due to its accelerated urbanization and industrialization [28], and increasing vehicles which have led to heavy traffic in cities [29], which is leading to increased levels of air pollutants. Arak is an industrialized city in central Iran and is one of the most polluted cities in the country, due to its heavy industrial activities, high number of motor vehicles, traffic and growing population.

Previous studies have reported associations between short-term air pollution exposure and respiratory deaths and hospitalization [13, 15, 17, 19, 20]. Most of these studies were conducted in developed countries, but, because of the susceptibility of different populations, different levels of ambient air pollutants, and characteristics of specific air pollutants, there is still a need to investigate the health effects of ambient air pollution exposure on human health in developing countries including Iran which there are few studies about the health impact of air pollutants especially in industrial cities such as Arak. This study aimed to investigate the short-term association between daily exposures to ambient air pollutants (NO_2_, CO, PM_2.5_, PM_10_, SO_2_ and O_3_) and respiratory hospital admissions in the urban population of Arak in a 6-year period.

## Methods

### Study area

This study was conducted in Arak, Iran which is the capital of the Markazi Province. Arak has a population of about 600,000 and includes 6 urban districts. It is located in the center of Iran and is about 288 km from Tehran. The total area of this city is 7178.98 km^2^ and it stands 1748 m above sea level. The weather of this city is relatively warm and dry in summer, and cold and humid in winter [30]. Its maximum temperature may raise up to 35 °C in summer and fall to below -15 °C in winter. The average annual rainfall is around 350 mm and the relative humidity is 46%. The annual average temperature is 13.9 °C. The geographic coordinates of this city are 34°5′30″ N and 49°41′21″ E. Arak is an active industrial city [31], and suffers from severe air pollution in the last decades, because of the presence of different emission sources, including industrial activities, increased number of motor vehicles and population growth [31–33].

### Data collection

This study was an ecological (population based) time series study. Air quality data was inquired from the archive of the Air Quality Monitoring Unit of the Arak Department of Environment from January 1^st^ 2010 to December 31^st^, 2015. Data prior to this period was not included due to the high percentage of missing data. Hourly air quality data is collected routinely in Arak in 4 fixed air pollution monitoring sites, located in the urban area of Arak. These stations routinely measure 6 air pollutants including CO, PM_2.5_, SO_2_, NO_2_, PM_10_ and O_3_. The location of these monitoring stations is not in the proximity of industrial polluters or major traffic sites and have sufficient distance from emitting sources.

In this study, we used the daily average concentrations of CO, PM_2.5_, SO_2_, NO_2_, PM_10_ and O_3_ (maximum 8-h moving average). If the concentration of a pollutant was not available in one monitoring station on a given day, the average values from the remaining stations were used to compute the average. The percent of missing values during the study period of 2191 days was 20% for CO, 25% for PM_10_, 30% for PM_2.5_, 32% for NO_2_, 34% for O_3_ and 35% for SO_2_. In this study, we imputed the missing air pollution data by using the EM algorithm method [34].

Meteorological data, including daily minimum, maximum, and average temperatures and minimum, maximum and average relative humidity, were obtained from the Arak Meteorological Organization for the same period.

Daily Hospital admissions were inquired for the same time period from two major hospitals (Amir-al-Momenin and Amir Kabir) in Arak. These two hospitals are governmental medical centers that admit people from various locations of this city. Another hospital in this city is the Qods private hospital which admits much less patients and has only 150 beds. The daily count of respiratory hospital admissions was aggregated by sex, date of hospital admission, age, and diagnosis according to the tenth revision of the International Classification of Diseases ((ICD-10) code J00-J99). The medical records information was extracted and was entered in standardized forms. We focused on the daily number of total hospital admissions occurring among the resident population in Arak city.

### Data analysis

A time-series regression analysis [35] was used to examine the short-term relationship between the count of respiratory admissions and air pollutants exposures (CO, NO_2_, O_3_, PM_2.5_, PM_10_ and SO_2_). This study used Generalized Linear Models (GLM) and Distributed Lag Models (DLM) within the family of Poisson distribution [35, 36], (Eq.1). We checked Poisson regression assumptions and because of its over dispersion we use quasi-Poisson regression models. In order to estimate the association between daily air pollutants and respiratory hospital admissions, the main exposure variable was the daily level of each individual air pollutant and the dependent variables were the daily counts of respiratory hospital admissions.

The Distributed Lag Model was used for lags up to 7 days (0–7 days), in order to evaluate the delayed effect of air pollutants [1, 36]. In order to control for seasonality and long-term trend in the data, a flexible spline function of time with 7 degrees of freedom (df) per year was used [35]. Also in order to adjust for the effects of temperature and relative humidity as potential confounders that change from day to day a natural cubic spline functions with 4 df was used for each [35–37]. The selection of degrees of freedom was based on minimizing Akaike’s Information Criterion (AIC). As almost all similar papers had used a number <10 for degrees of freedom, we tried 1 to 10 for the initial model and we used the number that had the lowest AIC for seasonality, long-term trend, temperature and relative humidity as the degrees of freedom (df).

Further, the day of the week (DOW) was also introduced into the model to adjust for the day of the week effect on hospital admissions. This variable shows the time interval from the previous holiday in days. Finally, we provide separate models for each pollutant, to reduce potential co-linearity between them [36]. The final model was described as below, (Eq. 1):

$$ \mathrm{Y}\mathrm{t} \sim \mathrm{Poisson}\ \left(\upmu \mathrm{t}\right) $$

$$ L n\left(\mu t\right)= a+{\displaystyle \sum_{t=0}^7\beta \iota AP}\iota + s\left( time,7\ast year\right)+ s\left( T,4 df\right)+ s\left( H,4 df\right)+\gamma DOW+ E $$

where, t refers to the day of the observation, Yt is the observed daily count of respiratory hospital admissions on day t, s denotes to a spline function, AP indicates the daily level of the air pollutants (PM_10_, NO_2_, CO, SO_2_, PM_2.5_ or O_3_), *ι* is the lag days, T is the average daily temperature, H is the average daily relative humidity. DOW is day of the week and E is error.

Lag terms were modelled separately and all together in unconstrained and constrained adjusted models. Additionally, sex (male, female) and age (under 60, and 60+ years) groups were modeled separately.

All statistical analyses were performed using R software version 3.3.1 (2016-06-21) (R Foundation for Statistical Computing, Vienna, Austria) [38] with time-series analyses using the “dlnm” package [39]. The results were presented as the Rate Ratio (RR) and its 95% confidence interval (CI) for daily respiratory hospital admissions, per 10 μg/m^3^ increase in each pollutant and per 1 mg/m^3^ increase in CO.

## Results

### Descriptive statistics

Summary statistics of respiratory hospital admissions, air pollutant concentrations, and meteorological data are provided in Table 1. The total number of respiratory hospital admissions for all ages were 15,622 during the study period and the daily mean count of respiratory admissions was 7.13. More than half (57.4%) of the respiratory hospital admissions were males, and the sex ratio was 1.35:1 (8966:6656) and 4843 (31%) of the respiratory admissions were in the elderly age group (60+ year-olds).

**Table 1:** Descriptive Statistics of air pollution levels, meteorological variables, and hospital admissions in Arak, 2010–2015

Variables	Mean ± SD	Minimum	25^th^ percentile	Median	75^th^ percentile	Maximum
O_3_ (μg/m^3^)	59.58 ± 26.7	1.5	41.47	55.97	72.82	186.03
CO (mg/m^3^)	2.89 ± 0.76	0.25	2.39	2.88	3.37	5.97
SO_2_ (μg/m^3^)	54.83 ± 33.3	1.59	37.49	47.87	61.91	566.85
PM_2.5_ (μg/m^3^)	24.3 ± 20.9	0.7	8.3	17.5	36.7	171.2
PM_10_ (μg/m^3^)	86.6 ± 44.3	2.3	62.1	82.04	99.3	536.3
NO_2_ (μg/m^3^)	53.45 ± 21.8	2.24	37.44	45.54	68.33	188.22
Temperature (°C)	14.8 ± 9.8	-15.1	6.7	15	23.9	33
Humidity (%)	44.9 ± 21.1	12	26	42	61	99
Respiratory admissions per day						
All	7.1 ± 5.5	0	3	6	10	38
Male	4.1 ± 3.4	0	1	3	6	23
Female	3.01 ± 2.7	0	1	2	5	22
0-18 year-olds	3.7 ± 3.9	0	0	3	6	21
19-60 year-olds	1.2 ± 1.4	0	0	1	2	18
60+ year-olds	2.2 ± 1.8	0	1	2	3	13

**Fig. 1:**
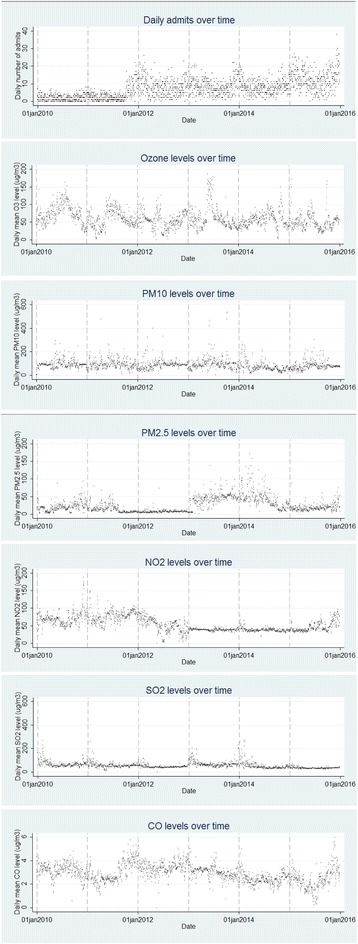
Daily total respiratory hospital admissions and air pollutants during the study period

Meanwhile, the daily average pollutant concentrations were 24.30 μg/m^3^ (from 0.7 to 171.21 μg/m^3^) for PM_2.5_ and 86.63 μg/m^3^ (from 2.3 to 536.28 μg/m^3^) for PM_10_ and these two concentrations were higher than the WHO 2014 guideline thresholds [40] which are 10 and 20 μg/m^3^ respectively (Table 1). The temporal pattern of air pollutants and daily total respiratory hospital admissions in the study period are showed in Fig. 1.

**Fig. 2:**
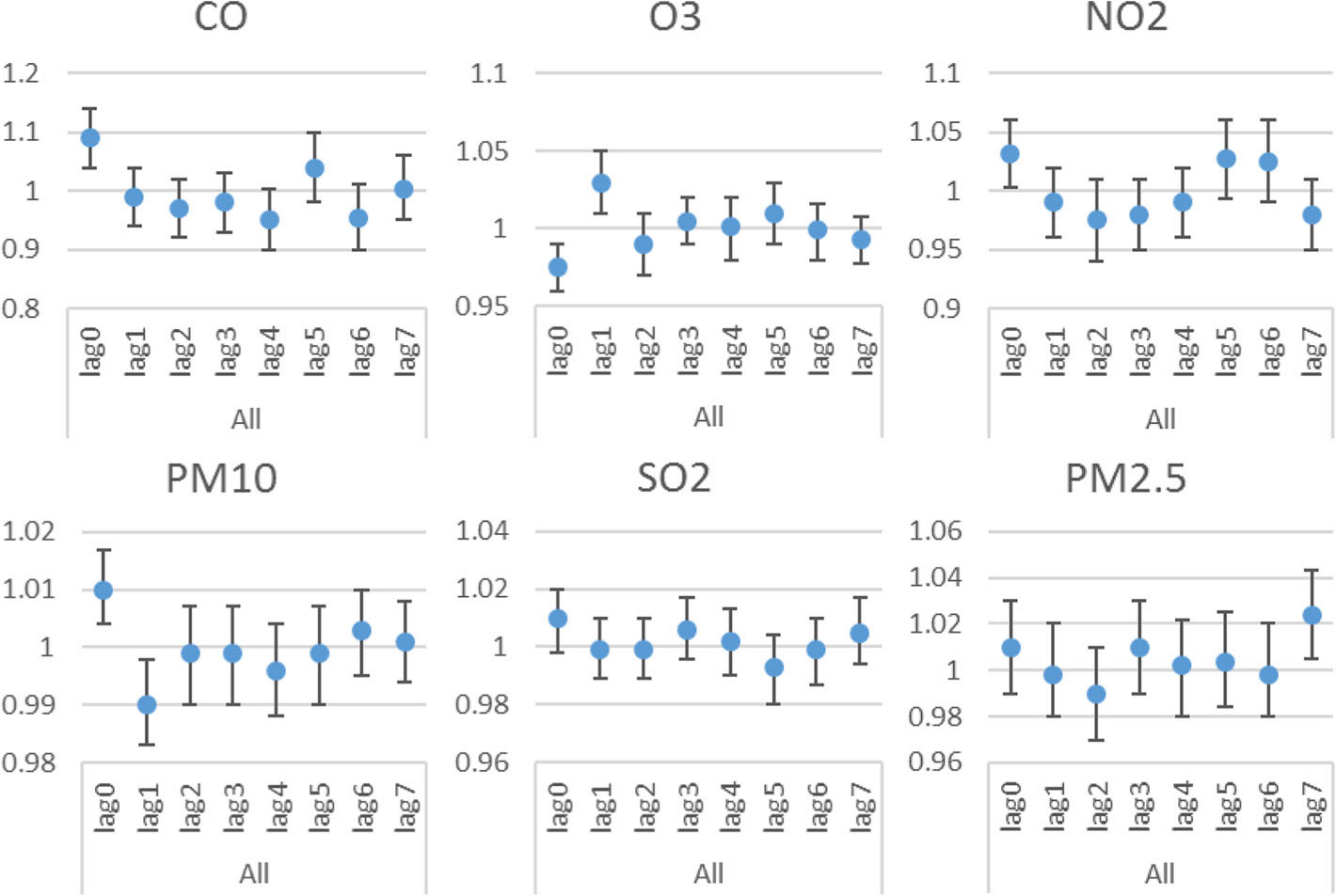
RRs (95% CIs) of respiratory admissions with an increase of 10 μg/m^3^ in air pollutants (and 1 mg/m^3^ in CO) according to adjusted unconstrained models

Table 2 and Fig. 2 shows the exposure-response relationship between air pollutants and respiratory hospital admissions for different lags and after adjustment for the long-term trend, weather conditions and DOW in single-pollutant models. Overall, the associations between each air pollutant and the number of respiratory hospital admissions were found to be significant for NO_2_ (*P* = 0.038), PM_10_ (*p* = 0.002) and CO (*p* = 0. 005), and the corresponding RRs and (95% CI) were 1.032(1.003-1.06), 1.01(1.004-1.017) and 1.09(1.04-1.14) per 10 μg/m^3^ increase in the concentrations of pollutants or 1 mg/m^3^ increase in CO, at lag 0 (day). O_3_ showed negative association with respiratory hospital admissions (*P* = 0.002), the corresponding RRs and (95% CI) were 0.975 (95%Cl: 0.96-0.99) per 10 μg/m^3^ increase at lag 0 day. Three air pollutants had lag effects, PM_10_ at lag 1 day, O_3_ at lag 1 day and PM_2.5_ at lag 7 day.

**Table 2:** RRs (95% CIs) of respiratory admissions with an increase of 10 μg/m^3^ in air pollutants (and 1 mg/m^3^ in CO) according to single lag, adjusted unconstrained and constrained DLM models for each air pollutant

Pollutant All:	Lag	Lag terms model one at a time RR (95% CI)	*p*-value	Adjusted unconstrained DLM RR (95% CI)	*p*-value	Adjusted constrained DLM RR (95% CI)	*p*-value
SO_2_	Lag 0	1.01(0.999-1.02)	0.06	1.01(0.998-1.02)	0.11	1.01(0.998-1.02)	0.12
	Lag 1	1.001(0.99-1.01)	0.74	0.999(0.989-1.01)	0.81	1.001(0.995-1.007)	0.87
	Lag 2	1.003 (0.994-1.012)	0.48	0.999 (0.989-1.01)	0.73	1.001(0.995-1.007)	0.87
	Lag 3	1.01(0.997-1.014)	0.22	1.006(0.996-1.017)	0.29	1.0005(0.997-1.004)	0.71
	Lag 4	1.003(0.995-1.01)	0.46	1.002(0.99-1.013)	0.40	1.0005(0.997-1.004)	0.71
	Lag 5	0.99(0.986-1.004)	0.26	0.993(0.98-1.004)	0.27	1.0005(0.997-1.004)	0.71
	Lag 6	0.996 (0.99-1.005)	0.42	0.999(0.987-1.01)	0.94	1.0005(0.997-1.004)	0.71
	Lag 7	1.0002(0.99-1.01)	0.96	1.005(0.994-1.017)	0.71	1.0005(0.997-1.004)	0.71
CO	Lag 0	1.07(1.02-1.12)	0.002	1.09(1.04-1.14)	0.005	1.09(1.04-1.14)	0.006
	Lag 1	0.997(0.955-1.04)	0.89	0.99(0.94-1.04)	0.39	0.975(0.95-1.005)	0.12
	Lag 2	0.96(0.92-0.999)	0.05	0.97(0.92-1.02)	0.43	0.975(0.95-1.005)	0.12
	Lag 3	0.95(0.90-0.997)	0.035	0.98(0.93-1.03)	0.55	0.984(0.97-0.998)	0.04
	Lag 4	0.95(0.91-0.995)	0.03	0.95(0.90-1.004)	0.07	0.984(0.97-0.998)	0.04
	Lag 5	0.99(0.94-1.04)	0.71	1.04(0.98-1.10)	0.19	0.984(0.97-0.998)	0.04
	Lag 6	0.95(0.91-0.994)	0.025	0.954(0.90-1.01)	0.08	0.984(0.97-0.998)	0.04
	Lag 7	0.98(0.94-1.02)	0.28	1.004(0.95-1.06)	0.97	0.984(0.97-0.998)	0.04
NO_2_	Lag 0	1.01(0.99-1.03)	0.29	1.032(1.003-1.06)	0.038	1.04(1.01-1.07)	0.011
	Lag 1	0.99(0.97-1.01)	0.45	0.99(0.96-1.02)	0.63	0.976(0.96-0.99)	0.01
	Lag 2	0.975(0.95-0.996)	0.02	0.975(0.94-1.01)	0.14	0.976(0.96-0.99)	0.01
	Lag 3	0.98(0.96-0.999)	0.04	0.98(0.95-1.01)	0.51	1.003(0.996-1.01)	0.40
	Lag 4	0.995(0.97-1.02)	0.63	0.99(0.96-1.02)	0.74	1.003(0.996-1.01)	0.40
	Lag 5	1.015(0.99-1.04)	0.16	1.027(0.993-1.06)	0.21	1.003(0.996-1.01)	0.40
	Lag 6	1.01(0.99-1.03)	0.30	1.025(0.99-1.06)	0.29	1.003(0.996-1.01)	0.40
	Lag 7	0.995(0.974-1.016)	0.66	0.98 (0.95-1.01)	0.27	1.003(0.996-1.01)	0.40
O_3_	Lag 0	0.995(0.98-1.01)	0.33	0.975 (0.96-0.99)	0.002	0.98(0.97-0.99)	0.008
	Lag 1	1.01(1.002-1.02)	0.02	1.03(1.01-1.05)	0.001	1.012(1.004-1.02)	0.01
	Lag 2	1.007(0.996-1.017)	0.20	0.99 (0.97-1.01)	0.31	1.012(1.004-1.02)	0.01
	Lag 3	1.01(1.0002-1.02)	0.046	1.005 (0.99-1.02)	0.52	1.001(0.997-1.004)	0.33
	Lag 4	1.01(0.998-1.02)	0.10	1.001 (0.98-1.02)	0.60	1.001(0.997-1.004)	0.33
	Lag 5	1.009(0.998-1.02)	0.09	1.01(0.99-1.03)	0.23	1.001(0.997-1.004)	0.33
	Lag 6	1.005(0.99-1.02)	0.39	0.999 (0.98-1.016)	0.99	1.001(0.997-1.004)	0.33
	Lag 7	1.0004(0.99-1.01)	0.94	0.993 (0.978-1.008)	0.29	1.001(0.997-1.004)	0.33
PM_2.5_	Lag 0	1.007(0.99-1.02)	0.46	1.01(0.99-1.03)	0.47	1.01(0.99-1.03)	0.37
	Lag 1	0.998(0.98-1.02)	0.89	0.998(0.98-1.02)	0.91	0.99 (0.98-1.002)	0.11
	Lag 2	0.99(0.97-1.01)	0.18	0.99(0.97-1.01)	0.16	0.99 (0.98-1.002)	0.11
	Lag 3	1.001(0.98-1.02)	0.92	1.01(0.99-1.03)	0.85	1.006(1.001-1.01)	0.03
	Lag 4	1.003(0.99-1.02)	0.71	1.002(0.98-1.022)	0.64	1.006(1.001-1.01)	0.03
	Lag 5	1.007(0.99-1.02)	0.39	1.004(0.984-1.025)	0.28	1.006(1.001-1.01)	0.03
	Lag 6	1.01 (0.99-1.03)	0.29	0.998 (0.98-1.02)	0.48	1.006(1.001-1.01)	0.03
	Lag 7	1.02(1.007-1.04)	0.005	1.024(1.005-1.043)	0.03	1.006(1.001-1.01)	0.03
PM_10_	Lag 0	1.004(0.999-1.01)	0.12	1.01(1.004-1.017)	0.002	1.01(1.002-1.02)	0.002
	Lag 1	0.995(0.99-1.001)	0.09	0.99(0.983-0.998)	0.02	0.995(0.99-0.998)	0.01
	Lag 2	0.995(0.99-1.001)	0.13	0.999(0.99-1.007)	0.95	0.995(0.99-0.998)	0.01
	Lag 3	0.997(0.99-1.003)	0.36	0.999(0.99-1.007)	0.85	1.001 (0.998-1.002)	0.57
	Lag 4	0.996(0.99-1.002)	0.25	0.996(0.988-1.004)	0.33	1.001 (0.998-1.002)	0.57
	Lag 5	0.999(0.99-1.005)	0.83	0.999(0.99-1.007)	0.61	1.001 (0.998-1.002)	0.57
	Lag 6	1.003(0.997-1.009)	0.31	1.003(0.995-1.01)	0.46	1.001 (0.998-1.002)	0.57
	Lag 7	1.004(0.998-1.01)	0.22	1.001(0.994-1.008)	0.70	1.001 (0.998-1.002)	0.57

**Fig. 3:**
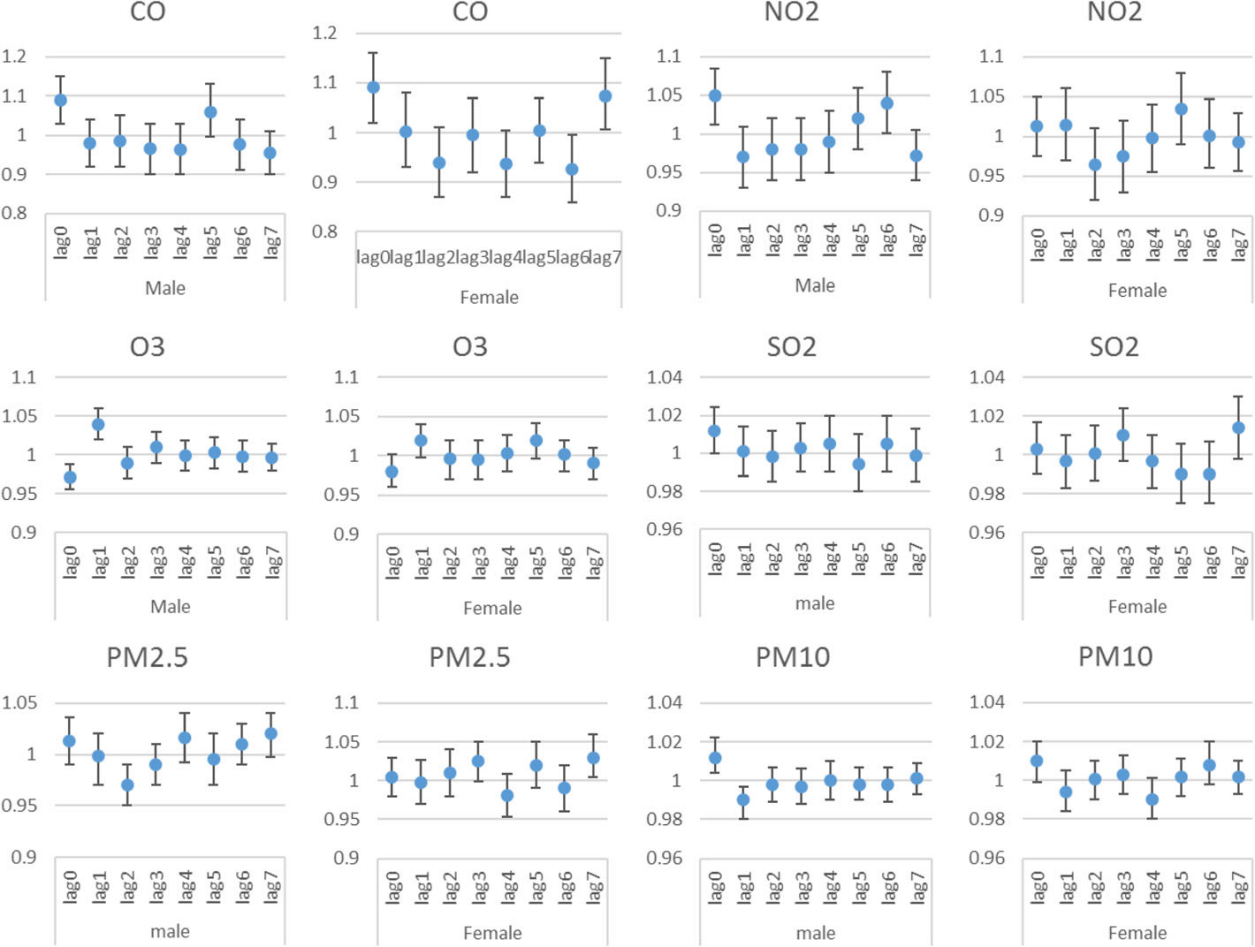
RRs (95% CIs) of respiratory admissions with an increase of 10 μg/m^3^ in air pollutants (and 1 mg/m^3^ in CO) according to adjusted unconstrained DLM models for each air pollutant in both genders

Table 3 and Fig. 3 show the exposure-response relationship between air pollutants and respiratory hospital admissions for every 10 μg/m^3^ increase in pollutant’s concentrations and every 1 mg/m^3^ increase in CO concentrations; for different lags, in single-pollutant models among different genders. Significant effects were observed for CO at lag-0 day (*P* = 0.026) and lag 7 day (*P* = 0.02), and PM_2.5_ at lag 7 day (*p* = 0.03) in females. Whereas males had a higher risk of respiratory admissions than females with an increase in PM_10_ at lag 0 day (*p* = 0.003), CO at lag 0 day (*p* = 0.02), SO_2_ at lag 0 day (*p* = 0.042), NO_2_ at lag 0 (*p* = 0.01) and at lag 6 days (*p* = 0.046), and O_3_ at lag 1 day (*P* = 0.001), in regard to respiratory hospital admissions.

**Table 3:** RRs (95% CIs) of respiratory admissions with an increase of 10 μg/m^3^ in air pollutants (and 1 mg/m^3^ in CO) according to single lag, adjusted unconstrained and constrained DLM models for each air pollutant in both genders

Pollutants		Lag	Lag terms model one at a time RR (95% CI)	*p*-value	Adjusted unconstrained DLM RR (95% CI)	*p*-value	Adjusted constrained DLM RR (95% CI)	*p*-value
Male	SO_2_	Lag 0	1.01(1.001-1.02)	0.04	1.012(1.0001-1.024)	0.042	1.01(1.001-1.02)	0.04
		Lag 1	1.003(0.992-1.013)	0.57	1.001(0.988-1.014)	0.77	1.0001(0.992-1.01)	0.91
		Lag 2	1.002(0.99-1.013)	0.65	0.998(0.985-1.012)	0.70	1.0001(0.992-1.01)	0.91
		Lag 3	1.002(0.991-1.012)	0.72	1.003(0.99-1.016)	0.74	1.001(0.997-1.005)	0.44
		Lag 4	1.005(0.99-1.015)	0.31	1.005(0.99-1.02)	0.21	1.001(0.997-1.005)	0.44
		Lag 5	0.998(0.987-1.009)	0.75	0.994(0.98-1.01)	0.47	1.001(0.997-1.005)	0.44
		Lag 6	1.0003(0.99-1.01)	0.96	1.005 (0.99-1.02)	0.56	1.001(0.997-1.005)	0.44
		Lag 7	0.998(0.987-1.009)	0.73	0.999(0.985-1.013)	0.87	1.001(0.997-1.005)	0.44
	CO	Lag 0	1.07(1.02-1.13)	0.007	1.09(1.03-1.15)	0.02	1.085(1.02-1.15)	0.03
		Lag 1	1.004 (0.95-1.06)	0.88	0.98(0.92-1.04)	0.32	0.98(0.94-1.02)	0.40
		Lag 2	0.98(0.93-1.03)	0.48	0.985(0.92-1.05)	0.98	0.98 (0.94-1.02)	0.40
		Lag 3	0.95(0.90-1.004)	0.07	0.965(0.90-1.03)	0.33	0.985(0.97-1.002)	0.053
		Lag 4	0.97(0.92-1.02)	0.25	0.964(0.90-1.03)	0.25	0.985(0.97-1.002)	0.053
		Lag 5	1.01(0.96-1.06)	0.63	1.06(0.995-1.13)	0.12	0.985(0.97-1.002)	0.053
		Lag 6	0.97(0.92-1.02)	0.19	0.976 (0.91-1.04)	0.51	0.985(0.97-1.002)	0.053
		Lag 7	0.945(0.898-0.994)	0.03	0.955(0.90-1.01)	0.06	0.985(0.97-1.002)	0.053
	NO_2_	Lag 0	1.018(0.993-1.044)	0.16	1.05(1.01-1.08)	0.01	1.048(1.014-1.08)	0.01
		Lag 1	0.99(0.965-1.017)	0.51	0.97(0.93-1.01)	0.21	0.97(0.95-0.99)	0.02
		Lag 2	0.98(0.955-1.006)	0.14	0.98(0.94-1.02)	0.50	0.97(0.95-0.99)	0.02
		Lag 3	0.98(0.956-1.006)	0.14	0.98(0.94-1.02)	0.58	1.004(0.996-1.012)	0.32
		Lag 4	0.995(0.97-1.02)	0.72	0.99(0.95-1.03)	0.69	1.004(0.996-1.012)	0.32
		Lag 5	1.017(0.99-1.044)	0.20	1.02(0.98-1.06)	0.39	1.004(0.996-1.012)	0.32
		Lag 6	1.018(0.993-1.045)	0.17	1.04(1.001-1.08)	0.046	1.004(0.996-1.012)	0.32
		Lag 7	0.995 (0.97-1.02)	0.70	0.972 (0.94-1.006)	0.13	1.004(0.996-1.012)	0.32
	O_3_	Lag 0	0.994(0.98-1.007)	0.35	0.972(0.956-0.988)	0.001	0.976(0.96-0.99)	0.008
		Lag 1	1.013(1.001-1.026)	0.035	1.04(1.02-1.06)	0.000	1.02(1.01-1.03)	0.008
		Lag 2	1.007(0.994-1.02)	0.30	0.99(0.97-1.01)	0.19	1.02(1.01-1.03)	0.008
		Lag 3	1.013(1.001-1.026)	0.034	1.01(0.99-1.03)	0.24	0.999(0.996-1.004)	0.71
		Lag 4	1.007(0.995-1.02)	0.28	0.999 (0.98-1.02)	0.73	0.999(0.996-1.004)	0.71
		Lag 5	1.005(0.99-1.02)	0.40	1.003(0.983-1.023)	0.77	0.999(0.996-1.004)	0.71
		Lag 6	1.002(0.99-1.01)	0.79	0.998 (0.978-1.018)	0.74	0.999(0.996-1.004)	0.71
		Lag 7	1.0001(0.99-1.01)	0.99	0.997 (0.98-1.014)	0.80	0.999(0.996-1.004)	0.71
	PM_2.5_	Lag 0	1.007(0.99-1.03)	0.50	1.013(0.99-1.036)	0.30	1.02(0.99-1.04)	0.2
		Lag 1	0.99 (0.97-1.01)	0.42	0.998(0.97-1.02)	0.91	0.98(0.97-0.99)	0.005
		Lag 2	0.97(0.95-0.99)	0.005	0.97(0.95-0.99)	0.014	0.98(0.97-0.99)	0.005
		Lag 3	0.98(0.96-1.004)	0.10	0.99(0.97-1.01)	0.23	1.005(0.998-1.01)	0.21
		Lag 4	1.003(0.98-1.02)	0.70	1.016(0.992-1.04)	0.15	1.005(0.998-1.01)	0.21
		Lag 5	0.997(0.98-1.01)	0.80	0.995(0.97-1.02)	0.92	1.005(0.998-1.01)	0.21
		Lag 6	1.01 (0.99-1.03)	0.35	1.01 (0.99-1.03)	0.82	1.005(0.998-1.01)	0.21
		Lag 7	1.02(1.002-1.04)	0.03	1.02(0.997-1.04)	0.15	1.005(0.998-1.01)	0.21
	PM_10_	Lag 0	1.005(0.998-1.01)	0.15	1.012(1.004-1.02)	0.003	1.01(1.003-1.02)	0.003
		Lag 1	0.99(0.98-0.999)	0.03	0.99(0.98-0.997)	0.01	0.992(0.988-0.997)	0.005
		Lag 2	0.99 (0.98-0.999)	0.03	0.998(0.989-1.007)	0.94	0.992(0.988-0.997)	0.005
		Lag 3	0.995 (0.99-1.002)	0.14	0.997(0.988-1.006)	0.31	0.999 (0.997-1.001)	0.66
		Lag 4	0.996(0.99-1.003)	0.30	1.0002(0.99-1.01)	0.99	0.999 (0.997-1.001)	0.66
		Lag 5	0.996(0.99-1.003)	0.30	0.998(0.99-1.007)	0.96	0.999 (0.997-1.001)	0.66
		Lag 6	0.998(0.99-1.005)	0.62	0.998(0.989-1.007)	0.91	0.999 (0.997-1.001)	0.66
		Lag 7	1.001(0.994-1.008)	0.80	1.001(0.993-1.009)	0.79	0.999 (0.997-1.001)	0.66
Female	SO_2_	Lag 0	1.005(0.994-1.016)	0.40	1.003(0.99-1.017)	0.32	1.002(0.99-1.01)	0.58
		Lag 1	0.999(0.988-1.01)	0.92	0.997 (0.983-1.01)	0.44	1.002(0.99-1.01)	0.87
		Lag 2	1.004(0.993-1.015)	0.48	1.001(0.987-1.015)	0.89	1.002(0.99-1.01)	0.87
		Lag 3	1.01(0.998-1.02)	0.09	1.01(0.997-1.024)	0.15	0.999(0.995-1.004)	0.80
		Lag 4	1.001(0.99-1.01)	0.91	0.997(0.983-1.01)	0.98	0.999(0.995-1.004)	0.80
		Lag 5	0.99(0.98-1.002)	0.11	0.99(0.975-1.006)	0.29	0.999(0.995-1.004)	0.80
		Lag 6	0.99(0.98-1.003)	0.13	0.99 (0.975-1.007)	0.43	0.999(0.995-1.004)	0.80
		Lag 7	1.003(0.99-1.014)	0.63	1.014(0.998-1.03)	0.41	0.999(0.995-1.004)	0.80
	CO	Lag 0	1.06(1.006-1.12)	0.03	1.09(1.02-1.16)	0.026	1.10(1.03-1.17)	0.02
		Lag 1	0.99(0.0.93-1.05)	0.68	1.002(0.93-1.08)	0.76	0.97(0.93-1.01)	0.08
		Lag 2	0.93(0.88-0.98)	0.009	0.94(0.87-1.01)	0.13	0.97(0.93-1.01)	0.08
		Lag 3	0.95(0.90-1.01)	0.12	0.995(0.92-1.07)	0.90	0.98(0.96-1.002)	0.18
		Lag 4	0.93(0.88-0.98)	0.014	0.937 (0.87-1.005)	0.08	0.98(0.96-1.002)	0.18
		Lag 5	0.965(0.91-1.02)	0.21	1.004(0.94-1.07)	0.69	0.98(0.96-1.002)	0.18
		Lag 6	0.93(0.88-0.99)	0.017	0.927 (0.86-0.995)	0.02	0.98(0.96-1.002)	0.18
		Lag 7	1.02(0.96-1.08)	0.52	1.074(1.006-1.15)	0.02	0.98(0.96-1.002)	0.18
	NO_2_	Lag 0	1.003(0.975-1.03)	0.83	1.013 (0.975-1.05)	0.41	1.024(0.99-1.06)	0.14
		Lag 1	0.993(0.965-1.02)	0.60	1.015(0.97-1.06)	0.57	0.98(0.96-1.003)	0.08
		Lag 2	0.97(0.942-0.996)	0.025	0.965(0.92-1.01)	0.08	0.98(0.96-1.003)	0.08
		Lag 3	0.975(0.95-1.002)	0.065	0.975(0.93-1.02)	0.64	1.002(0.99-1.01)	0.71
		Lag 4	0.994(0.97-1.02)	0.66	0.998(0.955-1.04)	0.89	1.002(0.99-1.01)	0.71
		Lag 5	1.013(0.986-1.04)	0.35	1.035(0.99-1.08)	0.22	1.002(0.99-1.01)	0.71
		Lag 6	1.003(0.975-1.03)	0.85	1.001(0.95-1.05)	0.84	1.002(0.99-1.01)	0.71
		Lag 7	0.996(0.97-1.02)	0.76	0.993 (0.956-1.03)	0.89	1.002(0.99-1.01)	0.71
	O_3_	Lag 0	0.995(0.98-1.01)	0.55	0.98(0.96-1.001)	0.09	0.985(0.97-1.004)	0.12
		Lag 1	1.011(0.997-1.025)	0.11	1.02(0.998-1.04)	0.11	1.007(0.996-1.02)	0.16
		Lag 2	1.007(0.993-1.022)	0.30	0.996 (0.97-1.02)	0.86	1.007(0.996-1.02)	0.16
		Lag 3	1.007(0.993-1.021)	0.32	0.995(0.97-1.02)	0.78	1.002 (0.997-1.006)	0.20
		Lag 4	1.011(0.997-1.025)	0.11	1.003 (0.98-1.026)	0.59	1.002 (0.997-1.006)	0.20
		Lag 5	1.015(1.0004-1.03)	0.044	1.02(0.996-1.042)	0.08	1.002 (0.997-1.006)	0.20
		Lag 6	1.008(0.995-1.02)	0.23	1.001 (0.98-1.02)	0.67	1.002 (0.997-1.006)	0.20
		Lag 7	1.001(0.987-1.016)	0.88	0.99 (0.97-1.01)	0.11	1.002 (0.997-1.006)	0.20
	PM_2.5_	Lag 0	1.006(0.98-1.03)	0.61	1.004(0.98-1.03)	0.98	1.004(0.98-1.03)	0.99
		Lag 1	1.01 (0.99-1.03)	0.45	0.998(0.97-1.026)	0.99	1.005(0.99-1.02)	0.58
		Lag 2	1.01 (0.99-1.03)	0.30	1.01(0.98-1.04)	0.61	1.005(0.99-1.02)	0.58
		Lag 3	1.02 (1.002-1.05)	0.03	1.025(0.999-1.05)	0.07	1.008(1.0003-1.015)	0.03
		Lag 4	1.003(0.98-1.03)	0.80	0.98(0.954-1.009)	0.31	1.008(1.0003-1.015)	0.03
		Lag 5	1.02(0.998-1.04)	0.064	1.02(0.99-1.05)	0.08	1.008(1.0003-1.015)	0.03
		Lag 6	1.01 (0.99-1.03)	0.46	0.99 (0.96-1.02)	0.13	1.008(1.0003-1.015)	0.03
		Lag 7	1.03(1.005-1.05)	0.016	1.03(1.005-1.06)	0.03	1.008(1.0003-1.015)	0.03
	PM_10_	Lag 0	1.004(0.996-1.01)	0.30	1.01(0.999-1.02)	0.32	1.006(0.997-1.01)	0.034
		Lag 1	0.998(0.99-1.006)	0.70	0.994(0.984-1.005)	0.24	0.997(0.99-1.003)	0.27
		Lag 2	0.999 (0.99-1.01)	0.90	1.0007(0.99-1.01)	0.98	0.997(0.99-1.003)	0.27
		Lag 3	1.0006 (0.99-1.01)	0.86	1.003(0.993-1.013)	0.38	1.001(0.999-1.004)	0.13
		Lag 4	0.996(0.99-1.005)	0.44	0.99(0.98-1.001)	0.09	1.001(0.999-1.004)	0.13
		Lag 5	1.003(0.995-1.01)	0.39	1.002 (0.992-1.011)	0.35	1.001(0.999-1.004)	0.13
		Lag 6	1.01(1.002-1.02)	0.015	1.008 (0.998-1.02)	0.14	1.001(0.999-1.004)	0.13
		Lag 7	1.007(0.999-1.014)	0.06	1.002(0.993-1.01)	0.71	1.001(0.999-1.004)	0.13

**Fig. 4:**
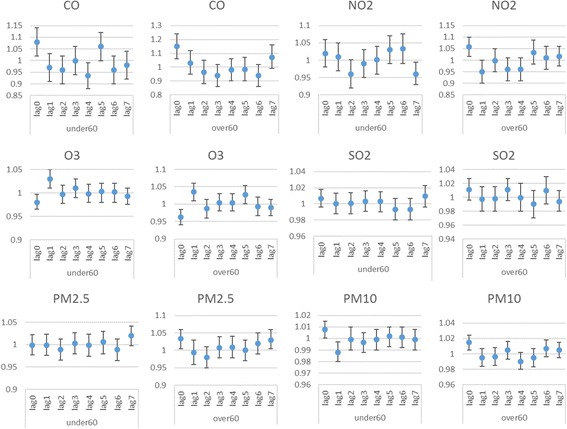
RRs (95% CIs) of respiratory admissions with an increase of 10 μg/m^3^ in air pollutants (and 1 mg/m^3^ in CO) according to adjusted unconstrained DLM models for each air pollutant among two age groups

Table 4 and Fig. 4 show the exposure-response relationship between air pollutants and respiratory hospital admissions for different lags, in single-pollutant models among different age groups. Positive and statistically significant associations were observed with NO_2_ (*P* = 0.005), PM_10_ (*p* = 0.009), PM_2.5_ (*P* = 0.03) and CO (*p* = 0.002) at lag 0 day, the corresponding RRs and (95% CI) were 1.06(1.02-1.10), 1.015(1.005-1.024), 1.03(1.005-1.06) and 1.15(1.06-1.024) per 10 μg/m^3^ increase in the concentrations of pollutants or 1 mg/m^3^ increase in CO in elderly (aged > 60) group. Negative significant associations were observed with O_3_ and respiratory hospital admissions (*P* = 0.001) in the elderly (aged > 60) group. The effect of CO, NO_2_ and PM_2.5,_ was strongest in the elderly (aged > 60) group. CO (*P* = 0.044) O_3_ (*P* = 0.004) and PM_10_ (*P *= 0.025) also showed positive significant association in aged < 60 years, group.

**Table 4:** RRs (95% CIs) of respiratory admissions with an increase of 10 μg/m^3^ in air pollutants (and 1 mg/m^3^ in CO) according to single lag, adjusted unconstrained and constrained DLM models for each air pollutant among two age groups

Pollutants		Lag	Lag terms model one at a time RR (95% CI)	*p*-value	Adjusted unconstrained DLM RR (95% CI)	*p*-value	Adjusted constrained DLM RR (95% CI)	*p*-value
Over60	SO_2_	Lag 0	1.01(0.998-1.023)	0.087	1.011(0.996-1.027)	0.10	1.01(0.995-1.023)	0.18
		Lag 1	1.002(0.99-1.015)	0.72	0.997(0.98-1.015)	0.83	1.001(0.99-1.01)	0.97
		Lag 2	1.005 (0.99-1.02)	0.43	0.998(0.98-1.015)	0.79	1.001(0.99-1.01)	0.97
		Lag 3	1.008(0.996-1.02)	0.20	1.01(0.995-1.027)	0.14	1.002(0.997-1.007)	0.49
		Lag 4	1.006(0.994-1.019)	0.31	0.999 (0.98-1.02)	0.63	1.002(0.997-1.007)	0.49
		Lag 5	0.999(0.986-1.012)	0.89	0.99(0.97-1.01)	0.45	1.002(0.997-1.007)	0.49
		Lag 6	1.001(0.99-1.01)	0.86	1.01 (0.99-1.03)	0.24	1.002(0.997-1.007)	0.49
		Lag 7	0.994(0.98-1.008)	0.43	0.994(0.98-1.01)	0.22	1.002(0.997-1.007)	0.49
	CO	Lag 0	1.13(1.06-1.20)	0.000	1.15 (1.06-1.24)	0.002	1.15 (1.07-1.24)	0.001
		Lag 1	1.05(0.98-1.12)	0.12	1.03(0.95-1.12)	0.50	0.985(0.94-1.03)	0.68
		Lag 2	0.976(0.91-1.04)	0.47	0.965 (0.88-1.05)	0.56	0.985(0.94-1.03)	0.68
		Lag 3	0.95(0.89-1.01)	0.15	0.94(0.86-1.02)	0.12	0.982(0.96-1.003)	0.10
		Lag 4	0.974(0.91-1.04)	0.44	0.98 (0.90-1.06)	0.58	0.982(0.96-1.003)	0.10
		Lag 5	0.97(0.91-1.03)	0.39	0.986 (0.90-1.07)	0.69	0.982(0.96-1.003)	0.10
		Lag 6	0.956(0.89-1.02)	0.19	0.94 (0.86-1.02)	0.26	0.982(0.96-1.003)	0.10
		Lag 7	1.03(0.96-1.10)	0.39	1.07(0.99-1.16)	0.11	0.982(0.96-1.003)	0.10
	NO_2_	Lag 0	1.003(0.975-1.03)	0.81	1.06 (1.02-1.10)	0.005	1.05 (1.01-1.09)	0.009
		Lag 1	0.97(0.94-0.998)	0.036	0.95 (0.90-1.001)	0.05	0.963(0.941-0.986)	0.001
		Lag 2	0.97(0.94-0.998)	0.03	0.998(0.95-1.05)	0.80	0.963(0.941-0.986)	0.001
		Lag 3	0.96(0.93-0.99)	0.009	0.96(0.91-1.01)	0.21	1.002(0.99-1.01)	0.47
		Lag 4	0.975(0.95-1.003)	0.08	0.96 (0.91-1.01)	0.27	1.002(0.99-1.01)	0.47
		Lag 5	1.005(0.98-1.03)	0.72	1.034(0.98-1.09)	0.32	1.002(0.99-1.01)	0.47
		Lag 6	1.01(0.98-1.04)	0.41	1.01(0.96-1.06)	0.54	1.002(0.99-1.01)	0.47
		Lag 7	1.01 (0.98-1.04)	0.36	1.016(0.975-1.06)	0.54	1.002(0.99-1.01)	0.47
	O_3_	Lag 0	0.98(0.97-0.99)	0.04	0.96(0.94-0.98)	0.001	0.97(0.95-0.99)	0.002
		Lag 1	1.01(0.99-1.02)	0.38	1.035(1.01-1.06)	0.02	1.01(0.999-1.02)	0.11
		Lag 2	0.999 (0.98-1.02)	0.99	0.987 (0.96-1.013)	0.38	1.01(0.999-1.02)	0.11
		Lag 3	1.01(0.99-1.03)	0.24	1.004(0.98-1.03)	0.73	1.003(0.998-1.008)	0.25
		Lag 4	1.01(0.997-1.03)	0.10	1.004 (0.98-1.03)	0.79	1.003(0.998-1.008)	0.25
		Lag 5	1.02(1.003-1.04)	0.017	1.027(1.001-1.054)	0.02	1.003(0.998-1.008)	0.25
		Lag 6	1.006(0.99-1.023)	0.46	0.993 (0.967-1.02)	0.31	1.003(0.998-1.008)	0.25
		Lag 7	0.998(0.98-1.015)	0.83	0.99(0.97-1.01)	0.54	1.003(0.998-1.008)	0.25
	PM_2.5_	Lag 0	1.03(1.002-1.05)	0.03	1.03(1.005-1.06)	0.03	1.04(1.007-1.06)	0.04
		Lag 1	1.004(0.98-1.03)	0.76	0.994 (0.96-1.03)	0.81	0.98 (0.97-1.001)	0.08
		Lag 2	0.99(0.97-1.02)	0.52	0.98 (0.95-1.01)	0.18	0.98 (0.97-1.001)	0.08
		Lag 3	1.01(0.99-1.04)	0.30	1.01(0.98-1.04)	0.86	1.01(1.005-1.02)	0.005
		Lag 4	1.02(0.99-1.04)	0.20	1.01(0.98-1.04)	0.58	1.01(1.005-1.02)	0.005
		Lag 5	1.02(0.99-1.05)	0.15	1.0002(0.97-1.03)	0.55	1.01(1.005-1.02)	0.005
		Lag 6	1.03 (1.006-1.06)	0.01	1.02 (0.99-1.05)	0.59	1.01(1.005-1.02)	0.005
		Lag 7	1.04(1.02-1.07)	0.001	1.03(1.004-1.06)	0.024	1.01(1.005-1.02)	0.005
	PM_10_	Lag 0	1.01(1.001-1.02)	0.03	1.015(1.005-1.024)	0.009	1.01(1.005-1.02)	0.01
		Lag 1	0.999(0.99-1.008)	0.88	0.995(0.984-1.007)	0.45	0.995(0.99-1.002)	0.27
		Lag 2	0.996(0.99-1.005)	0.40	0.996(0.985-1.008)	0.64	0.995(0.99-1.002)	0.27
		Lag 3	0.998(0.99-1.007)	0.70	1.005(0.99-1.02)	0.45	1.0003 (0.997-1.003)	0.94
		Lag 4	0.99 (0.98-1.002)	0.12	0.99 (0.98-1.002)	0.12	1.0003 (0.997-1.003)	0.94
		Lag 5	0.996(0.99-1.004)	0.31	0.993 (0.98-1.007)	0.58	1.0003 (0.997-1.003)	0.94
		Lag 6	1.006(0.997-1.014)	0.16	1.007(0.996-1.02)	0.38	1.0003 (0.997-1.003)	0.94
		Lag 7	1.006(0.998-1.014)	0.13	1.005(0.995-1.015)	0.48	1.0003 (0.997-1.003)	0.94
Under60	SO_2_	Lag 0	1.007(0.997-1.017)	0.16	1.007(0.996-1.02)	0.25	1.008(0.997-1.02)	0.21
		Lag 1	1.002(0.99-1.012)	0.74	1.0005(0.988-1.013)	0.95	1.001(0.99-1.01)	0.93
		Lag 2	1.002(0.99-1.01)	0.64	1.001(0.988-1.014)	0.80	1.001(0.99-1.01)	0.93
		Lag 3	1.004(0.994-1.014)	0.45	1.004(0.99-1.02)	0.68	0.999(0.995-1.004)	0.97
		Lag 4	1.001(0.99-1.01)	0.82	1.003 (0.99-1.015)	0.45	0.999(0.995-1.004)	0.97
		Lag 5	0.99(0.98-1.003)	0.18	0.993(0.98-1.007)	0.35	0.999(0.995-1.004)	0.97
		Lag 6	0.993(0.98-1.005)	0.25	0.993(0.98-1.007)	0.41	0.999(0.995-1.004)	0.97
		Lag 7	1.003(0.99-1.014)	0.57	1.01(0.996-1.023)	0.22	0.999(0.995-1.004)	0.97
	CO	Lag 0	1.05(1.001-1.10)	0.04	1.08(1.02-1.14)	0.044	1.07(1.02-1.03)	0.036
		Lag 1	0.98(0.93-1.03)	0.40	0.97(0.91-1.03)	0.14	0.97(0.94-1.002)	0.075
		Lag 2	0.95(0.90-1.0002)	0.051	0.96(0.90-1.02)	0.50	0.97(0.94-1.002)	0.075
		Lag 3	0.96(0.91-1.01)	0.10	0.999(0.94-1.06)	0.88	0.986(0.97-1.002)	0.11
		Lag 4	0.94(0.89-0.99)	0.02	0.934(0.88-0.99)	0.03	0.986(0.97-1.002)	0.11
		Lag 5	1.003(0.95-1.05)	0.89	1.06(0.998-1.12)	0.054	0.986(0.97-1.002)	0.11
		Lag 6	0.95(0.90-1.001)	0.051	0.96(0.90-1.02)	0.12	0.986(0.97-1.002)	0.11
		Lag 7	0.96(0.91-1.01)	0.10	0.98(0.92-1.04)	0.54	0.986(0.97-1.002)	0.11
	NO_2_	Lag 0	1.02(0.99-1.05)	0.17	1.02(0.98-1.06)	0.22	1.03(1.0004-1.06)	0.04
		Lag 1	1.006(0.98-1.03)	0.63	1.01 (0.97-1.05)	0.61	0.98(0.96-1.002)	0.18
		Lag 2	0.98(0.95-1.01)	0.14	0.96(0.92-1.002)	0.09	0.98(0.96-1.002)	0.18
		Lag 3	0.99(0.96-1.02)	0.48	0.99(0.95-1.03)	0.86	1.004(0.996-1.012)	0.40
		Lag 4	1.01(0.98-1.04)	0.56	1.002(0.96-1.04)	0.97	1.004(0.996-1.012)	0.40
		Lag 5	1.024(0.997-1.05)	0.08	1.03(0.99-1.07)	0.33	1.004(0.996-1.012)	0.40
		Lag 6	1.01(0.98-1.04)	0.35	1.033(0.99-1.076)	0.23	1.004(0.996-1.012)	0.40
		Lag 7	0.983(0.956-1.009)	0.20	0.96(0.93-0.995)	0.045	1.004(0.996-1.012)	0.40
	O_3_	Lag 0	0.999(0.988-1.01)	0.99	0.98(0.965-0.997)	0.035	0.985(0.97-1.001)	0.09
		Lag 1	1.02(1.01-1.03)	0.01	1.03(1.01-1.05)	0.004	1.01(1.003-1.02)	0.01
		Lag 2	1.01(0.999-1.02)	0.07	0.997(0.978-1.016)	0.52	1.01(1.003-1.02)	0.01
		Lag 3	1.01(0.999-1.02)	0.054	1.01(0.99-1.03)	0.47	0.999(0.996-1.003)	0.50
		Lag 4	1.006(0.994-1.018)	0.29	0.998(0.98-1.018)	0.59	0.999(0.996-1.003)	0.50
		Lag 5	1.005(0.993-1.017)	0.40	1.003(0.98-1.02)	0.82	0.999(0.996-1.003)	0.50
		Lag 6	1.004(0.992-1.016)	0.50	1.002 (0.98-1.02)	0.53	0.999(0.996-1.003)	0.50
		Lag 7	1.001(0.99-1.01)	0.86	0.993(0.976-1.01)	0.26	0.999(0.996-1.003)	0.50
	PM_2.5_	Lag 0	0.997(0.98-1.02)	0.75	0.999(0.98-1.02)	0.84	1.001(0.98-1.02)	0.93
		Lag 1	0.996(0.98-1.02)	0.68	0.999 (0.98-1.02)	0.99	0.99 (0.98-1.01)	0.28
		Lag 2	0.99(0.97-1.006)	0.16	0.99(0.97-1.01)	0.25	0.99 (0.98-1.01)	0.28
		Lag 3	0.994(0.97-1.01)	0.59	1.003(0.98-1.03)	0.93	1.003(0.996-1.01)	0.33
		Lag 4	0.997(0.98-1.02)	0.81	0.999(0.97-1.02)	0.69	1.003(0.996-1.01)	0.33
		Lag 5	1.002(0.98-1.022)	0.84	1.006(0.98-1.03)	0.31	1.003(0.996-1.01)	0.33
		Lag 6	0.999 (0.98-1.02)	0.91	0.989(0.96-1.01)	0.25	1.003(0.996-1.01)	0.33
		Lag 7	1.02(0.997-1.04)	0.10	1.02(0.997-1.04)	0.22	1.003(0.996-1.01)	0.33
	PM_10_	Lag 0	1.002(0.99-1.01)	0.51	1.008(1.0004-1.015)	0.025	1.01(0.999-1.015)	0.17
		Lag 1	0.99 (0.98-0.999)	0.04	0.988(0.98-0.997)	0.01	0.994(0.99-0.998)	0.008
		Lag 2	0.99 (0.987-1.001)	0.12	0.999(0.99-1.01)	0.95	0.994(0.99-0.998)	0.008
		Lag 3	0.997(0.99-1.003)	0.31	0.996(0.99-1.005)	0.38	1.0001 (0.998-1.002)	0.45
		Lag 4	0.998 (0.99-1.005)	0.60	0.999(0.99-1.008)	0.85	1.0001 (0.998-1.002)	0.45
		Lag 5	1.001(0.99-1.01)	0.75	1.002 (0.993-1.01)	0.30	1.0001 (0.998-1.002)	0.45
		Lag 6	1.002(0.995-1.01)	0.62	1.001(0.992-1.01)	0.61	1.0001 (0.998-1.002)	0.45
		Lag 7	1.002(0.996-1.01)	0.49	0.999(0.99-1.008)	0.96	1.0001 (0.998-1.002)	0.45

**Fig. 5:**
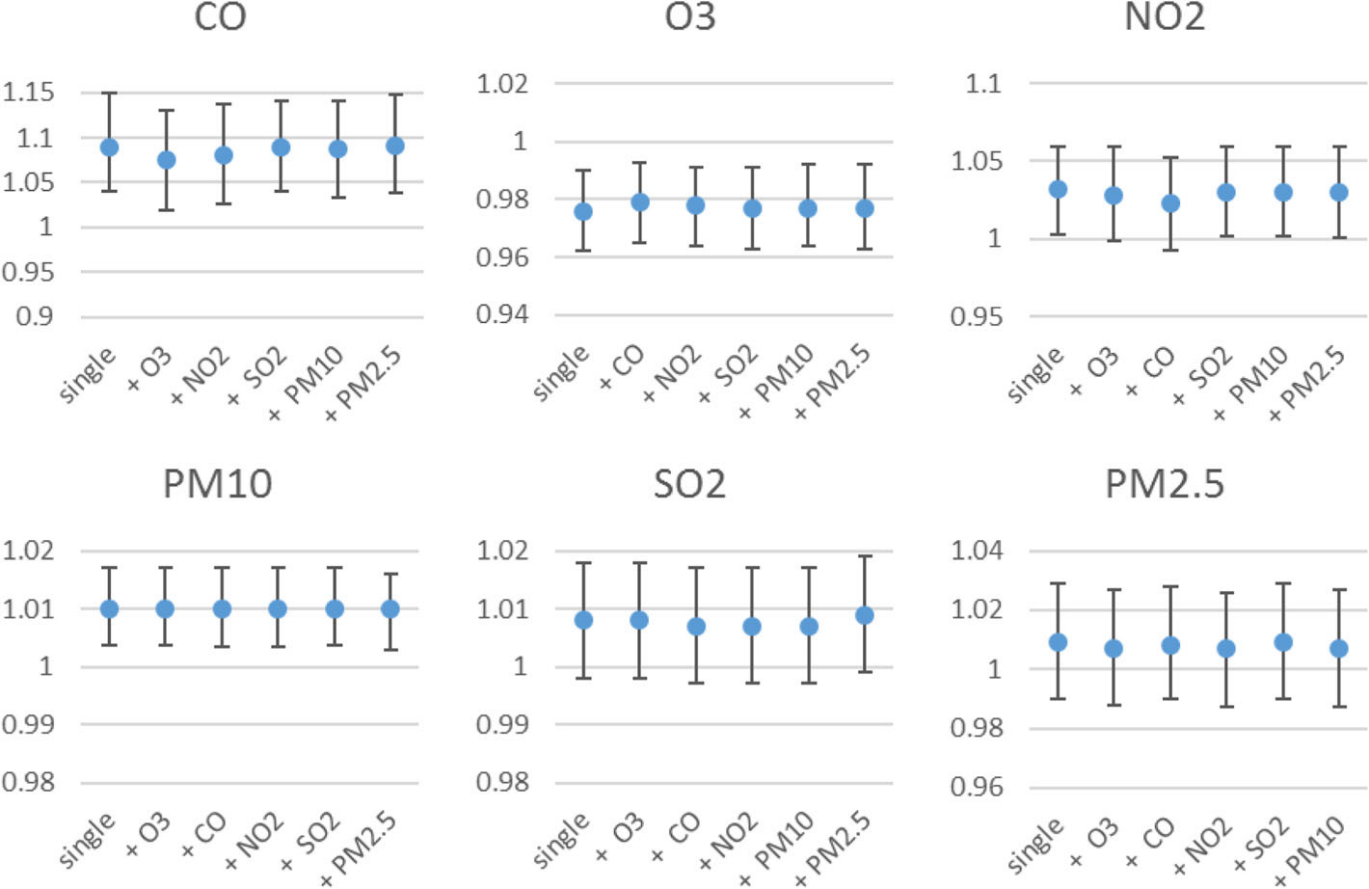
RRs (95% CIs) of respiratory admissions with an increase of 10 μg/m^3^ in air pollutants (O_3_, NO_2_, PM_10_, SO_2_, PM_2.5_, and an increase of 1 mg/m^3^ in CO) in two-pollutant models

Fig. 5 shows the exposure-response relationship between air pollutants and respiratory hospital admissions for every 10 μg/m^3^ increase in pollutant’s concentrations and every 1 mg/m^3^ increase in CO concentrations; in two-pollutant models. Almost all exposure-response relationship between air pollutants and respiratory hospital admissions were relatively constant after adjusting for other air pollutants. The association between respiratory hospital admissions and SO_2_ tended to be significant after adjustment for PM_2.5_ (RR = 1.009, 95%Cl: 0.999-1.019), but not other pollutants. As for NO_2_, when adjusted for CO and O_3_, the estimated effect decreased to (RR = 1.023, 95%Cl: 0.993-1.053) and (RR = 1.028, 95%Cl: 0.999-1.06), respectively and were insignificant.

## Discussion

The present study showed that, CO, PM_10_ and NO_2_ had a positive association with respiratory hospital admissions. However, the association between other pollutants including SO_2_ and PM_2.5_ and daily hospital respiratory admissions was only significant in males and the elderly at lag 0 day, respectively. Unexpectedly, O_3_ had a negative association with respiratory hospital admissions.

PM_10_ has been shown to produce oxidative stress and increase inflammatory markers in exposed subjects [41]. In this study, the effect estimate was 1% (95%CI: 1.004-1.017), increase in respiratory hospital admissions per 10 μg/m^3^ increase in PM_10_. Several studies have suggested that ambient PM_10_ is effective on respiratory hospital admissions. For example, a study by Atkinson et al in 2001 investigated the adjusted short-term health effects of ambient particles, in eight European cities, and found a 0.9% increased risk of total respiratory disease hospitalizations for each 10 μg/m^3^ increase in PM_10_ [42]. Ma et al in 2016 in Lanzhou, China estimated the risk of emergency room visits for respiratory diseases associated with exposure to ambient air pollution in the spring dust storm season and concluded that ER visits for respiratory diseases increased by 1.14% for each 10 μg/m^3^ increase in PM_10_ at lag-3 day [21]. Other studies [43] from Korea, [13] the US, [9] Italy and [20] China, reported that a 10 μg/m^3^ increase in PM_10_ was associated with a 0.77%, 3.2%, 0.77% and 0.2% increase in hospitalization for respiratory diseases. In contrast to this study, Shahi et al in 2014 reported no increase in respiratory diseases hospitalizations related to PM_10_ in Tehran, Iran. However, this insignificancy can be due to the short study period (2012-2013) and use of limited data. In Shahi’s study data from only one hospital was used. Meanwhile, the mean annual concentration of PM_10_ was 64.72 μg/m^3^ [23] which was less than the current study and Tao [20] (196.63 μg/m^3^) and Ma [21] (159.2 μg/m^3^) studies.

In this study, no positive association was found between O_3_ or PM_2.5_ levels and respiratory hospitalizations on the same day (at lag 0 day). Also, we observed a protective effect of O_3_ on respiratory hospitalizations after adjustment for other air pollutants. But the lag model showed that ozone had a significant adverse effect on respiratory admissions at lag 1 day. A study done by Wilson et al from Europe in 2005 reported that increases in O_3_, was not positively associated with the number of all respiratory hospitalizations in Portland and Manchester, UK [19]. Another study done by Phung et al investigated air pollution and the risk of respiratory and cardiovascular hospitalizations in Vietnam and did not show any significant association between O_3_ and respiratory hospitalizations [36]. Also Fung et al study in 2005 in Ontario, Canada did not find any association between O_3_ and respiratory diseases admissions either [6]. On the other hand, some studies have suggested a negative effect of ozone on respiratory hospital admissions [13, 44, 45]. Altogether, the inconsistency in effects of ozone on respiratory diseases admissions may be dependent on its concentration or patient’s characteristics (such as age, sex, occupation or poverty) [46]. For example, despite the higher ozone concentration during summer, more people use air conditioners that may reduce the effect of ozone on health. However, more studies are needed to clarify these contradictory results.

The results of this study were also comparable to Slaughter et al study from the US, that did not find a significant associated between PM_2.5_ levels and respiratory hospital admissions in Spokane, Washington [47] either. However, PM_2.5_ has shown significant associations with respiratory hospital admissions in some other studies. Xu et al explored the association between fine particulate air pollution (PM_2.5_) and respiratory hospital emergency room visits in Beijing, China and found a positive association between them at different lags [48]. Zanobetti et al in 26 US communities explored the association between fine particulate air pollution (PM_2.5_) and cause-specific respiratory emergency admissions and found a 10 μg/m^3^ increase in 2-day averaged PM_2.5_concentration was associated with a 2.07% increase in respiratory admissions [17]. Also Dominic et al’s study in 204 US counties showed a significant positive association between PM_2.5_ and respiratory hospital admissions [8]. The association between PM_2.5_ and daily hospital respiratory admissions in older adults (60+ year-olds) was significant at lag-0 in the present study where the mean annual concentration of PM_2.5_ was equal to 24.3 μg/m^3^ and is consistent with Xu et al’s study with mean annual concentration of PM_2.5_ equal 102.1 μg/m^3^ [48] from China.

In the current study, CO showed a significant association with respiratory hospital admissions. Several other world studies are in line with these results [15, 23, 49]. Samoli et al’s study in London, UK reported evidence for a consistent adverse effects of short-term CO exposures on adult respiratory hospital admissions [49]. In another study done by Shahi et al in 2014, in Tehran, Iran; total respiratory diseases hospitalizations increased by 4% for each 10 μg/m^3^ increase in CO levels in urban areas [23]. A quantitative systematic review including 134 papers and estimates from 173 cities also resulted in a significant association between the CO levels and respiratory hospital admissions [50]. However, a study by Slaughter et al from the US, found no significant association between CO levels and respiratory hospital admissions in Spokane, Washington [47].

The results of this study were comparable to Chen et al study, in 2010 from China, that did not find a significant association between SO_2_ levels and respiratory hospital admissions in Shanghai [51]. Also another study done by Shahi et al in Tehran, Iran did not show an increase in respiratory diseases hospitalizations with increases in SO_2_ concentrations [23] either. In these two mention studies, the mean annual concentration of SO_2_ were 56 μg/m^3^ [51] and 32.22 μg/m^3^ [23], respectively; which are equal or lower than the SO_2_ concentrations in our study (54.83 μg/m^3^). However, these insignificant results can be due to the short study durations (2005-2007) in Chen et al and (2012-2013) in Shahi et al’s two studies and also use of a small sample size. On the other hand, SO_2_ has shown significant associations with respiratory hospital admissions in some other studies. For example research from Lanzhou, China reported that ER visits for respiratory diseases increased by 2.7% for each 10 μg/m^3^ increase in SO_2_ on dust days and by 0.6% for each 10 μg/m^3^ increase in SO_2_ on non-dust days [21]. Also other studies from China such as, Zhang et al’s study from Beijing, [1], Tao et al’s study from Lanzhou [20], and Liu et al’s study from Jinan [22], reported that an increase of 10 μg/m^3^ of SO_2_ corresponded to a 35%, 0.5% and 1.2% increase of respiratory diseases hospitalizations, respectively. Another study done by Phung et al from Vietnam also found that SO_2_ was positively associated with the number of respiratory hospital admissions [36]. The mean annual concentration of SO_2_ in most of the mentioned studies that reported significant association between SO_2_ levels and respiratory hospital admissions, were higher than the present study. This concentration was reported, 79.09 μg/m^3^ in Lanzhou, China [20], 95.4 μg/m^3^ in Jinan, China [22], and 79.1 μg/m^3^ in western China [52], that almost all of them have, 1.5 to 2 times the SO_2_ concentration of the present study. However, in our study the association between SO_2_ and daily hospital respiratory admissions in males was significant, despite the relatively low concentration of SO_2_.

NO_2_ is a highly reactive oxidant which contributes to increased susceptibility to respiratory infections [53]. The main sources of ambient NO_2_ are industrial emissions and motor vehicle exhaust in Arak. The association between NO_2_ and daily hospital respiratory admissions in this study is consistent with previous studies. Liu et al in Jinan, China showed that an increase of 10 μg/m^3^ of NO_2_ corresponded to a 2.5% increase of respiratory disease hospitalizations [22]. Another study in Tehran, Iran found that total respiratory diseases hospitalizations were increased by 1% for each 10 μg/m^3^ increase in NO_2_ level [23]. A study from Lanzhou, China also reported that total respiratory disease hospitalizations were increased by 11.0% for each 10 μg/m^3^ increase of NO_2_ on dust days and by 2.5% for 10 μg/m^3^ increases in NO_2_ on non-dust days [21]. Tao et al in Lanzhou, China, found that total respiratory disease hospitalizations increased by 1.1% for each 10 μg/m^3^ increase in NO_2_ levels [20]. In contrast, a previous study from Europe found no significant associations between NO_2_ and respiratory diseases hospitalizations. In the mentioned study, the mean daily concentrations of NO_2_ was 50.3 ppb [54]. Another study done by Rezaei et al in 2016, did not find a significant association between NO_2_ concentrations and respiratory disease hospitalizations in Kerman, Iran. The mean annual concentrations of NO_2_ was very low and equal to 0.04 ppm in the Kerman study [55], which is lower than the present study that the mean annual concentrations of NO_2_ was 53.45 μg/m^3^.

Several studies investigated the effects of air pollution on human health, in single-pollutant models, among different gender and age groups [13, 15, 21, 36, 49]. In this study, there were different health effects of air pollution between males and females in regard to respiratory admissions. Some studies observed different health effects of air pollution between two genders. In the current study, significant adverse effects were observed for CO at lag 0 and lag7 day and PM_2.5_ at lag-7 in females, while a higher risk of respiratory hospital admissions was seen in males for PM_10_ at lag 0, CO at lag 0, SO_2_ at lag 0, NO_2_ at lag 0 and lag 6 day, and O_3_ at lag 1 days. These results could be related to men’s occupation in jobs such as industry or taxi driving or more outdoor activities which expose them to more air pollution[56, 57]. This study also found that older adults (60+ year-olds) were more vulnerable to respiratory disease exacerbations. These findings are consistent with previous studies that the elderly were more susceptible to exposure to outdoor air pollution [1, 20, 21].

One of the limitations of this study was missing air pollutant data. Missing data is a frequent problem in many scientific fields, especially in studies about the effects of ambient air pollutants [34, 58]. Missing data is common in air quality monitoring stations due to unpredicted technical malfunctions or faulty equipment, that effect data storage [34]. There are three types of missing data according to their generation, including missing completely at random (MCAR), missing at random (MAR) and missing not at random (MNAR) [59, 60]. In the present study missing data occurred due to failure of air quality monitoring stations and it was not related to the pollutant levels in certain days.

There are many methods for dealing with missing data. For example, complete case analysis is the default method used by most statistical software that exclude incomplete observations from analysis. But under the MCAR and MAR assumption, if a high proportion of incomplete observations are excluded, loss of precision may happen [34]. Also in time series analysis, excluding this observations by using the complete case method may impair the temporal pattern of the data, including trends, seasonality and autocorrelation [34, 61]. In this study, the missing data imputed by using the expectation-maximization (EM) algorithm method [34].

However, additional analysis was also done by using complete case analysis. Result of complete case analysis generally showed the same results but with lower precision compared to EM imputed data. However, the effect of SO_2_ at lag 0 (RR, 95% CI = 1.02, (1.006-1.035)) and PM_2.5_ at lag 0 (RR, 95% CI = 1.03, (1.006-1.055)) was significant in complete case analysis, but not in EM algorithm imputed data. These findings further emphasize the negative effects of air pollutants in Arak.

The other limitations of this study were that in ecological studies, such as the current study, the results cannot be directly inferred to individual levels. Another limitation of this study was limiting the cases to two major hospitals in Arak. Also, we were not able to control potential individual confounders, such as smoking, genetic susceptibility or migrations, and population displacements.

## Conclusion

The results of this investigation show that some outdoor air pollutants were associated with increased respiratory hospital admissions. The strongest association was seen for CO and NO_2_. This study also found evidence that males and elderly age groups are more susceptible to air pollutants. These findings suggest new evidence about the health effects of air pollutions, in the Middle East region, which despite its increased motor vehicles, traffic and industrialization has not yet adopted appropriate strategies to control air pollution.

## Abbreviations

CI: Confidence interval
CO: Carbon monoxide
Df: Degrees of freedom
DLM: Distributed Lag Models
DOW: Day of the week
ER: Emergency room
GLM: Generalized linear models
ICD: International classification of diseases
IQR: Interquartile range
NO_2_: Nitrogen dioxide
O_3_: Ozone
PM_10_: Particulate matter 10 micrometers or less in diameter
PM_2.5_: Particulate matter 2.5 micrometers or less in diameter
Ppb: Parts per billion
RR: Rate Ratio
SO_2_: Sulfur dioxide
